# Hypertonic saline improves renal oxygenation, renal function and inflammation following ischemia/reperfusion-induced acute kidney injury

**DOI:** 10.1186/2197-425X-3-S1-A457

**Published:** 2015-10-01

**Authors:** B Ergin, C Ince

**Affiliations:** Academic Medical Center, Amsterdam, the Netherlands

## Introduction

The pathogenesis of AKI is characterized by a deterioration in tissue perfusion and oxygenation in combination with inflammation resulting in renal microcirculatory dysfunction and kidney failure. Hypertonic saline (HS) solution increases serum osmolarity which results in an redistribution of fluid from the interstitial and intracellular spaces to the intravascular space potentially improving tissue perfusion (1). It has been suggested that HS has also anti-inflammatory effects (2).

OBJECTIVE

In this study, we hypothesized that hemodynamic and inflammatory effects of HS may protect the kidney by promoting renal microcirculatory oxygenation and function following ischemia/reperfusion in rat kidney.

METHOD

24 mechanically ventilated Wistar albino rats with a mean ± SD body weight of 250-350 g under deep anesthesia were studied. Rats were divided into 4 groups (n = 6/group): (1) control group; (2) a group subjected to renal ischemia for 45 min by supra-aortic occlusion followed by 2 h of reperfusion (I/R); and (3-4) I/R groups treated with an continuous i.v. infusion 5 ml/kg/h either Normal Saline (IR+NS) or Hypertonic Saline (10% NaCl) (I/R+HS) after ischemia. Systemic and renal hemodynamic, renal cortical and medullar microcirculatory pO_2_ as well as renal function parameters and inflammation markers were assessed.

## Results

MAP values reduced in IR group at T1 (p < 0.05), were significantly elevated by treatment of HS at T2 (p < 0.01) with respect to the IR group. Renal vascular resistance (RVR) was elevated in the NS group (p < 0.001) with respect to the Control and I/R group, but not in the HS group. cmPO2, mmPO_2_, renal oxygen deliver (DO_2ren_) and consumption (VO_2ren_) levels were improved in I/R group received HS but not with NS. HS caused a decrease in TNa^+^ correlated with an elevation of fractional sodium excretion (EFNa^+^) and urine output. TNF-a, IL-6 and hyaluronic acid levels in renal tissue samples of the HS group were significantly lower than the I/R and I/R+NS group (p < 0.05).

## Conclusions

I/R induced AKI causes a deterioration of renal microcirculatory oxygenation, oxygen supply and consumption associated with an increase levels of inflammatory mediators and hemodynamic instability. The renal protective effects of HS in this study may be due to improved systemic hemodynamic, microcirculatory perfusion and oxygenation, or its anti-inflammatory and diuretic effect associated with an increased Na excretion, or combination of these effects.

## Grant Acknowledgment

This study was supported in part by a grant from the Dutch Kidney Foundation (grant C 09.2290).
